# Spectroscopic Investigation of Thioacrolein by Variational and Perturbational Approaches

**DOI:** 10.1002/jcc.70343

**Published:** 2026-04-08

**Authors:** Guntram Rauhut

**Affiliations:** ^1^ Institute for Theoretical Chemistry University of Stuttgart Stuttgart Germany

**Keywords:** rotational transitions, thioacrolein, vibrational configuration interaction, vibrational perturbation theory, vibrational transitions

## Abstract

The vibrational spectra of *trans* and *cis*‐thioacrolein have been studied by 2nd order vibrational perturbation theory (VPT2) and vibrational configuration interaction (VCI) theory. All calculations rely on multilevel potential energy surfaces (PESs) with the highest level being explicitly correlated coupled‐cluster theory including single and double excitation and a perturbational treatment of the triple excitation in combination with an augmented triple‐ζ basis set. Comparisons with experimental data are provided, including a number of predictions for unobserved fundamentals. Moreover, (ro) vibrational configuration interaction (RVCI) theory has been used for the simulation of the microwave spectrum, accompanied by the calculation of spectroscopic constants from perturbation theory.

## Graphical abstract

The vibrational and rotational spectra of trans and cis‐thioacrolein were studied by high levels of electronic and vibrational structure theory. Comparisons with experimental results are provided for several molecular quantities.

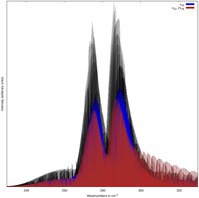

## 1 Introduction

While many studies in the literature deal with acrolein, investigations focusing on thioacrolein are scarce. Searching the Web of Science^1^ for acrolein resulted in 8636 hits, but only in 49 hits for thioacrolein (or 2‐propenethial). With that, comparably little is known about this molecule. The reason for that is its instability caused by dimerization to vinyldithiin via a Diels‐Alder reaction, in which the molecule can act as both the diene and the dienophile. Nevertheless, it has been subject to spectroscopic investigations after its generation by a pyrolysis of diallyl sulfide. For example, Judge and Moule [1] measured its photoelectron spectrum, and Korolev and Baskir [2] recorded an infrared spectrum. Georgiou and Kroto [3] studied the microwave spectrum of this molecule, and most recently, Cabezas et al. [4] detected it in the interstellar medium toward TMC‐1 based on rotational spectroscopy laboratory experiments. These authors were also able to determine a full set of quartic and sextic centrifugal distortion constants. As sulfur chemistry in the instellar medium is an active field of current research, the observation of this molecule in space moves this molecule more into the focus of interest and motivated the present study.

With respect to quantum chemical calculations, even less has been done. Early studies were based on Hartree‐Fock calculations only, while the recent study of Cabezas et al. [4] employed coupled‐cluster calculations, that is, CCSD(T). However, accurate quantum chemical calculations at this level or even beyond for the vibrational and rotational spectrum, including a full account of anharmonicity, are completely missing. Therefore, a comprehensive study of its infrared and microwave spectra by explicitly correlated electronic structure methods and (ro)vibrational configuration interaction (RVCI) theory is considered a timely task and augments available experimental information about this molecule [5].

Thioacrolein shows two conformers, a *trans* and a *cis*‐conformer, with the *trans*‐conformer being the more stable one. In most experiments, signals of both conformers can be seen. In principle, an anharmonic frequency study would require a multidimensional potential energy surface (PES) including both minima and the connecting transition state. However, this results in a computationally very demanding task and was therefore beyond the available possibilities. Consequently, the local PESs of both minima have been computed independently, introducing a considerable approximation.

## 2 Computational Details

The equilibrium geometries of *cis* and *trans*‐thioacrolein have been determined at two levels of electronic structure theory. The first one is based on frozen core (fc) explicitly correlated coupled‐cluster theory including single and double excitations and a perturbational treatment of the triple excitations [6] in combination with an augmented triple‐ζ orbital basis set [7], that is, fc‐CCSD(T)‐F12b/cc‐pVTZ‐F12. The second level includes core‐correlation effects and estimated high‐order coupled‐cluster terms based on Goodson's continued fraction approach [8]. While the sole consideration of core‐correlation effects usually leads to a worse representation of the vibrational frequencies, the additional inclusion of correction terms as obtained from an extrapolation of the coupled‐cluster energies was found to yield much more balanced results [9, 10]. As a consequence, the basis set had to be enlarged by tight core functions for these all‐electron (ae) calculations, that is ae‐CCSD(T)‐F12b/cc‐pCVTZ‐F12 [11]. Note that, the use of triple‐ζ basis sets in combination with explicitly correlated methods leads to results of similar accuracy as obtained from conventional methods based on a quintuple‐ζ basis [12]. Harmonic frequencies and normal coordinates have been determined at these levels for both conformers.

Normal coordinates were employed to span the multidimensional PESs. An n‐mode expansion [13] truncated after the 4‐mode coupling terms has been used to reduce the number of electronic structure calculations. Moreover, multilevel schemes [14, 15] have been exploited to limit the computational effort, that is, while the 1D and 2D terms were determined at the same level as used for the harmonic frequency calculations, the 3D terms were determined from explicitly correlated distinguishable cluster calculations including single and double excitations [16] and a basis set of double‐ζ quality, that is, DCSD‐F12b/cc‐pVDZ‐F12. The 4D terms were calculated at the MP2/cc‐pVDZ level. Within the construction of the PES using ae methods, core‐correlation effects and the inclusion of approximate high‐order coupled‐cluster terms were only realized for the 1D‐3D terms, while the 4D terms were again based on fc‐MP2 calculations. The PESs have been generated in a fully automated manner, exploiting symmetry and intermediate fitting [17]. In total $1.2\cdot 10^{6}$ (1D: 112; 2D: 6982; 3D 192,832; 4D: 1,004,080 grid points) ab initio calculations have been performed to generate grid representations of the PESs.

Dipole moment surfaces (DMS) have exclusively been determined from conventional calculations as analytical expressions for the explicitly correlated methods have not yet been implemented into the molpro package [18], which has been used for all calculations of this study. The 1D and 2D dipole surfaces were obtained from DCSD/cc‐p(C)VTZ‐F12 calculations, the 3D terms from DCSD/cc‐p(C)VDZ‐F12 calculations, and the 4D terms again from MP2/cc‐pVDZ calculations. As conventional electronic structure methods converge more slowly with respect to the basis set in comparison to explicitly correlated methods, basis set effects for the dipole surfaces must be expected to be stronger than for the corresponding PESs, but are expected to be sufficiently small for this study. The grid representations of the PESs and DMSs have been transformed to analytical ones by means of Kronecker product fitting [19], which exploits a sum‐of‐products structure within the n‐mode expansion. Up to 12 monomials per coordinate have been employed, which was found to be sufficient in order to yield negligible least‐squares fitting errors of less than $10^{-10}$ $E_{h}^{2}$.

Optimized one‐mode vibrational wavefunctions (modals) were obtained from state‐specific vibrational self‐consistent field (VSCF) calculations [20] based on the Watson Hamiltonian [21]. A basis of 18 distributed Gaussians has been employed for this task. Within the VSCF iterations, a constant μ‐tensor, that is, the inverse of the moment of inertia tensor at the equilibrium geometry, has been used within the calculation of the vibrational angular momentum terms during the VSCF iterations. The modals of the vibrational ground state have been used to generate Hartree products (configurations) for the subsequent configuration selective vibrational configuration interaction (VCI) calculations [22, 23] neglecting any rotations (J=0). Up to sextuple excitations have been considered in these calculations, but the correlation space was further restricted by the sum of quantum numbers (up to 12) and the maximal excitation per oscillator (up to the 9th root). This led to about $10^{7}$ configurations per irrep. Vibrational angular momentum terms using an n‐mode expansion of the μ‐tensor being truncated after 1st order were added to the diagonal elements of the VCI matrix, while 0th order terms were added to the off‐diagonal terms only. For details of the configuration selection process and further aspects of this program, see Refs. [22, 23, 24].

11 low‐lying VCI wavefunctions including overtones and combination bands have been used as a vibrational basis in subsequent RVCI calculations for the simulation of the rotational spectrum. These states were limited to at most triple excitations. Wang combinations [25] of rigid rotor functions were used as rotational basis functions. The maximal rotational quantum number J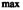 was set to 135. Symmetry was used within the construction of the real‐valued RVCI matrices [26]. The expansion of the μ‐tensor was truncated after the 2nd order terms within the purely rotational part of the Watson operator. This resulted in a line list with more than 4.5 million entries, which was used for a comparison with experimental data and to simulate the rotational spectrum. Due to the C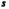 symmetry of both conformers, the nuclear spin statistical weights for both irreducible representations of this point group are the same and were determined to be 16.

## 3 Geometrical Parameters

**FIGURE 1:**
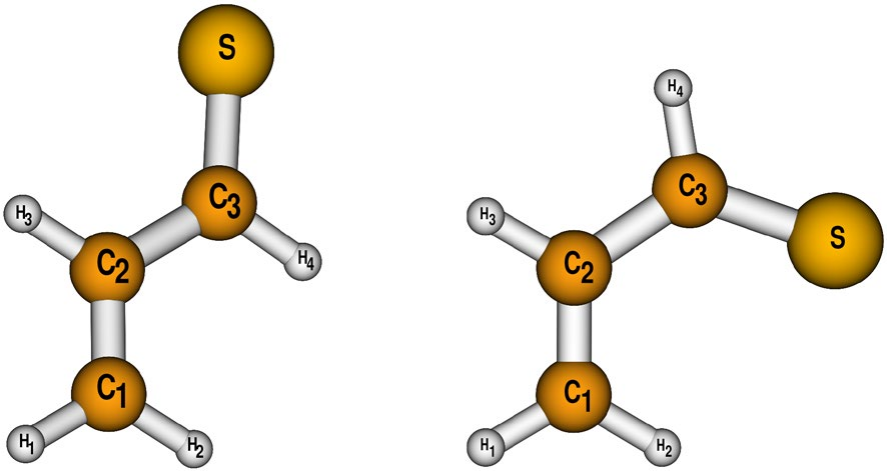
Structures and atomic labeling of *trans* and *cis*‐thioacrolein.

According to the fc‐CCSD(T)‐F12b/cc‐pVTZ‐F12 calculations, the *trans*‐conformer is 2.6 kcal/mol more stable than the *cis*‐conformer, both of them being displayed in Figure 1. This value does not alter upon the inclusion of core correlation effects. The rotational barrier was determined to be 8.2 kcal/mol (2884 ${\text{cm}}^{-1}$) with respect to the *trans*‐conformer.

The computed geometrical parameters are summarized in Table 1. ${\text{r}}_{e}$ denotes equilibrium parameters as obtained from the individual geometry optimizations, while ${\text{r}}_{a}$ and ${\text{r}}_{g}$ characterize vibrationally averaged bond lengths [27]. The averaging was based on the VCI wavefunction of the vibrational ground state, that is, temperature effects have not been considered. While ${\text{r}}_{a}$ denotes an averaging of the atomic positions, ${\text{r}}_{g}$ has been obtained from the expectation values of the respective bond lengths.

**TABLE 1:** Computed geometrical parameters of *trans* and *cis*‐thioacrolein.

Coord.	trans‐C 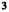 H 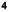 S						cis‐C 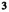 H 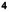 S					
	fc			ae			fc			ae		
	$r_{e}$	$r_{a}$	$r_{g}$	$r_{e}$	$r_{a}$	$r_{g}$	$r_{e}$	$r_{a}$	$r_{g}$	$r_{e}$	$r_{a}$	$r_{g}$
r(C 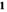 C 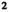 )	1.3438	1.3463	1.3514	1.3412	1.3438	1.3489	1.3416	1.3454	1.3492	1.3390	1.3427	1.3467
r(C 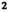 C 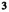 )	1.4526	1.4601	1.4617	1.4493	1.4567	1.4584	1.4663	1.4707	1.4751	1.4631	1.4672	1.4718
r(C 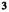 S)	1.6263	1.6286	1.6315	1.6224	1.6247	1.6276	1.6270	1.6299	1.6322	1.6231	1.6259	1.6283
r(C 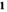 H 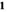 )	1.0815	1.0834	1.1019	1.0801	1.0820	1.1005	1.0812	1.0814	1.1016	1.0799	1.0799	1.1002
r(C 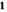 H 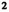 )	1.0836	1.0866	1.1043	1.0823	1.0852	1.1029	1.0831	1.0799	1.1037	1.0819	1.0780	1.1023
r(C 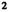 H 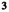 )	1.0837	1.0894	1.1048	1.0824	1.0882	1.1036	1.0844	1.0855	1.1055	1.0831	1.0838	1.1042
r(C 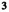 H 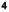 )	1.0908	1.0965	1.1122	1.0895	1.0952	1.1109	1.0890	1.0897	1.1104	1.0877	1.0880	1.1091
∠(C 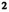 C 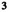 S)	124.75			124.77			126.82			126.81		
∠(C 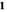 C 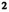 C 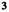 )	121.44			121.48			124.14			124.15		
∠(SC 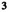 H 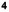 )	119.92			119.90			118.43			118.42		
∠(C 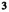 C 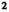 H 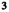 )	117.06			117.05			116.04			116.04		
∠(C 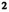 C 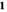 H 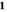 )	121.57			121.57			120.92			120.95		
∠(C 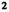 C 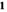 H 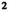 )	120.82			120.82			120.52			120.49		

*Note:* Bond lengths are given in Å and angles in degree. The PES was determined by frozen core and all electron/continued fraction explicitly correlated coupled‐cluster calculations.

Clearly, ${\text{r}}_{g}$ bond lengths are consistently longer than ${\text{r}}_{a}$ bonds and both are longer than the corresponding equilibrium values, ${\text{r}}_{e}$, as must be expected from anharmonicity. As repeatedly been discussed in the literature [28], core correlation effects lead to shorter bond lengths in comparison to the fc approximation, but the estimated inclusion of high‐order coupled‐cluster terms diminishes this effect slightly. The ae results are considered to be the best values in this table. Experimental values have been provided by Georgiou and Kroto [3], but these authors provide ${\text{r}}_{s}$ values, which have been obtained from isotopic substitution and which are thus not directly comparable with the values provided in Table 1. They determined the length of the C=C bond to 1.341±0.009 Å, the C‐C bond to be 1.455±0.027 Å, and the C=S bond to be 1.61±0.02 Å. These values are in slightly better agreement with the ae than the fc results, but the accuracy is too low to allow for any further conclusions.

## 4 Vibrational Frequencies

Independent VCI calculations have been performed for the *trans* and the *cis*‐conformer. As many correlating functions are energetically above the low‐lying rotational barrier (see above), the independent treatment must be considered a considerable approximation, which inevitably will lead to certain inaccuracies. However, a common treatment of both minima in one calculation would require other coordinates than normal coordinates and our current VCI implementation, based on the Podolsky Hamiltonian [29], which in principle allows for arbitrary coordinates, is not yet efficient enough in order to handle a system of this size. A comparison with the experimental matrix isolation data (Ar) of Korolev and Baskir [2] was possible for *trans*‐C
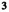
H
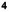
S and several bands of *cis*‐C
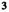
H
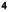
S. One needs to keep in mind that measurements in an Ar matrix usually lead to deviations with respect to gas phase data arising from cavity effects and the rather strong polarizability of the Ar atom. It has been shown in several studies that these deviations are slightly larger than those of accurate VCI results, which may easily amount to more than 10 ${\text{cm}}^{-1}$ [30, 31]. Besides that, Georgiou and Kroto [3] determined the low‐lying $\nu_{18}$ and $\nu_{13}$ bands of *trans*‐C
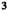
H
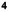
S from the vibrational satellite assignments in the microwave spectrum of *trans*‐thioacrolein. However, these authors estimated the uncertainty of these bands to be ±30 ${\text{cm}}^{-1}$. The VCI results, as well as results from 2nd order vibrational perturbation theory (VPT2), are summarized in Table 2 for *trans*‐thioacrolein and in Table 3 for *cis*‐thioacrolein.

**TABLE 2:** Vibrational frequencies [${\text{cm}}^{-1}$] and infrared intensities [km/mol] of *trans*‐thioacrolein obtained from VCI and VPT2 calculations.

Sym	#	Harm. (fc)	VPT2		VCI				Exp.
			$\nu_{i}$ (fc)	IR (fc)	$\nu_{i}$(fc)	IR (fc)	$\nu_{i}$ (ae)	IR (ae)	
A 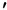	1	3244.2	3097.4	6.5	3098.2	1.3	3102.2	4.1	3108.5
	2	3183.4	3022.2	1.0	3026.8	0.9	3030.8	1.5	2989.3^a^
	3	3146.0	2975.5	2.1	2982.0	3.1	2981.4	2.0	2989.3^a^
	4	3091.9	2943.1	2.6	2950.5	5.2	2954.7	4.7	2723.0^a^
	5	1643.7	1596.9	3.1	1603.1	4.7	1607.0	4.1	1598.0
	6	1461.8	1423.0	36.3	1422.5	27.9	1424.8	40.2	1424.2
	7	1332.4	1303.5	8.0	1304.4	7.7	1306.4	7.7	1305.8
	8	1300.6	1275.8	7.1	1274.4	6.8	1276.6	6.4	1279.1
	9	1194.7	1169.2	19.5	1169.4	22.7	1172.1	22.8	1173.7
	10	1089.6	1074.5	20.0	1074.0	20.7	1076.6	21.3	1071.8
	11	898.4	886.7	0.3	885.3	0.3	887.6	0.3	890.0
	12	453.1	451.2	0.2	450.9	0.2	452.6	0.2	(450)^a^
	13	278.1	279.5	2.3	279.6	3.4	280.7	3.4	280^a^
A 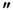	14	1018.3	992.7	17.2	992.6	18.7	994.0	17.4	1001.9
	15	957.7	941.4	38.0	936.3	35.5	937.9	35.5	943.0
	16	891.2	878.9	3.8	878.3	3.8	880.8	3.6	—
	17	621.9	616.8	8.1	615.4	6.8	616.9	6.7	553.5
	18	144.2	141.2	0.1	141.1	0.1	141.6	0.1	140^a^

^a^ See the discussion.

**TABLE 3:** Vibrational frequencies [${\text{cm}}^{-1}$] and infrared intensities [km/mol] of *cis*‐thioacrolein obtained from VCI and VPT2 calculations.

Sym	#	Harm. (fc)	VPT2		VCI				Exp.
			$\nu_{i}$ (fc)	IR (fc)	$\nu_{i}$ (fc)	IR (fc)	$\nu_{i}$ (ae)	IR (ae)	
A 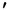	1	3250.4	3102.9	3.2	3107.1	1.0	3110.2	1.2	
	2	3170.6	3014.1	7.4	3026.8	6.2	3032.3	2.5	
	3	3148.7	3049.9	6.2	2971.2^a^	0.1	3031.4	4.6	
	4	3105.4	2973.9	9.7	2962.3	4.5	2966.3	4.7	
	5	1648.4	1609.3	2.8	1609.1	2.7	1612.1	3.1	
	6	1436.3	1394.8	6.2	1397.3	21.4	1398.6	21.6	1396.5
	7	1390.2	1355.4	21.4	1358.1	20.0	1359.2	20.3	1355.0
	8	1323.9	1298.0	5.9	1299.2	6.3	1300.8	6.2	1297.6
	9	1155.3	1135.4	13.8	1136.0	6.9	1140.6	9.7	1136.8
	10	1021.5	1008.1	16.5	1012.3	8.9	1013.6	9.0	
	11	878.4	865.3	3.9	870.4	3.7	869.9	4.0	
	12	562.9	553.9	5.3	555.7	5.0	557.7	4.9	
	13	255.5	239.5	0.7	250.8	0.6	247.9	0.3	
A 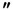	14	1008.6	984.8	20.3	980.2	5.6	981.9	4.6	
	15	973.9	959.0	17.7	953.6	18.3	955.2	19.7	954.9
	16	882.0	871.5	8.2	874.6	8.4	875.5	9.2	871.0
	17	568.2	549.4	15.7	546.1	17.6	549.2	16.2	
	18	82.8	109.0	0.7	107.5	0.7	104.7	0.7	

^a^ See the discussion.

### 4.1 *trans*‐Thioacrolein

The overall agreement of the computed data, irrespective of whether VPT2 or VCI, fc or ae, with the experimental data is good. However, several transitions need to be discussed explicitly, either because the deviation with respect to the experimental reference data is huge or due to problems within the calculations. Let us consider the CH‐stretching modes first. Apparently, Korolev and Baskir assigned a band at 2989.3 ${\text{cm}}^{-1}$ to both, the $\nu_{2}$ and $\nu_{3}$ fundamentals, which is very unlikely and which is not supported by any of the calculations. While the calculations for $\nu_{3}$ agree well with this value, a much higher value for $\nu_{2}$ has been obtained. However, the VCI calculations indicate strong vibrational couplings, which lead to extremely low leading VCI coefficients, that is, 0.51 for $\nu_{2}$ and also for $\nu_{3}$ (fc). With that, a clear state identity is lost, and an assignment to a fundamental or a coupling combination band is questionable and error‐prone. Besides a questionable assignment, this indicates a strong sensitivity of these bands, and thus matrix isolation effects can be significant. However, VCI calculations agree reasonably well with the VPT2 results, which show large resonance corrections. Moreover, Korolev and Baskir assigned a band at 2723.0 ${\text{cm}}^{-1}$ to the $\nu_{4}$ fundamental. The deviation relative to the calculations is more than 200 ${\text{cm}}^{-1}$, and thus this is considered a misassignment. This fundamental shows a 1‐3 Darling‐Dennison resonance with the $\nu_{7}+\nu_{9}+\nu_{12}$ combination band. This pair splits up in two transitions at 2950.5 and 2956.8 ${\text{cm}}^{-1}$ (fc). Although the difference between the fc‐VPT2 results and the corresponding ae‐VCI values are as large as 11.6 ${\text{cm}}^{-1}$, these values are considered to be more accurate than the experimental assignment.

The agreement of the calculations with the experimental reference data in the fingerprint region is excellent with one notable exception. Korolev and Baskir assigned $\nu_{17}$ to a band a 553.5 ${\text{cm}}^{-1}$. According to both the VPT2 and VCI calculations, this fundamental shows a strong type‐2 Fermi resonance with the $\nu_{12}+\nu_{18}$ combination band. This indicates again a strong sensitivity and thus matrix isolation effects may have a strong impact on this Fermi pair, for which the lower eigenvalue was computed at 588.0 (fc) and 589.8 ${\text{cm}}^{-1}$ (ae). With that, this is not considered to be a misassignment, but indicates a strong cavity effect. For all transitions in the fingerprint region, the agreement between the different calculations is very good and shows typical trends, for example, the ae transitions are slightly blue‐shifted in comparison to the fc results. The good agreement of the VPT2 results with the fc‐VCI results arises from the rigidity of this molecule, indicating also small contributions from the high‐order terms of the PES. As the determination of a quartic force field as needed for the VPT2 calculation is less demanding than the evaluation of the more accurate n‐mode expansion for the VCI calculations, a restriction to it appears to be a good approximation for this particular molecule.

Despite the huge error bar, which Georgiou and Kroto assigned to their band origins of $\nu_{18}$ and $\nu_{13}$, their values are in excellent agreement with the calculations. Moreover, these authors assigned the band origin of the corresponding combination band $\nu_{13}+\nu_{18}$ to 450±30 ${\text{cm}}^{-1}$. According to the ae calculations, this transition is much lower, that is, 424.8 ${\text{cm}}^{-1}$, but, as can be seen from Table 2, the value of Georgiou and Kroto agrees extremely well with the results for the fundamental of $\nu_{12}$. Consequently, the question arises whether the determined band origin belongs to this fundamental rather than the combination band. For that reason, the experimental value in Table 2 has been put into parentheses.

Judge and Moule [1] were able to deduce a sequence of overtones for the torsional mode, that is, $\nu_{18}$, $2\nu_{18}$, $3\nu_{18}$ and $4\nu_{18}$, from their photoelectron measurements. They obtained these transitions at 140.8, 280.7, 419.8, and 558.0 ${\text{cm}}^{-1}$. The first value is in excellent agreement with the result of Georgiou and Kroto and the values in Table 2. The respective overtones were computed at the ae‐VCI level at 280.4, 421.7, and 564.1 ${\text{cm}}^{-1}$. While the $2\nu_{18}$ and $3\nu_{18}$ overtones match nicely the results of Judge and Moule, the result for the $4\nu_{18}$ transition is somewhat higher. These results indicate that the curvature of our potential in high‐energy regions is not as good described as in regions close to the equilibrium geometry which arises from the low‐lying rotational barrier, that is, the potential is strongly curved, but due to the fact that the barrier is not fully included in the multidimensional PES, the fitting procedure leads to polynomials, which deviate from the exact potential. In order to resolve this problem, one would need to switch to internal coordinates and to describe both minima at the same time (*vide supra*) as has been done quite recently for the HOPO molecule [32]. Within that study, it was found that treating the conformers separately had hardly any impact on all other fundamentals than the torsional one, but the influence on the ladder of torsional overtones was significant. This was not so much due to tunneling effects, but a proper description of the PES. However, the result of this study, that the sequence of overtones of the torsion is well described for the four lowest transitions of thioacrolein, indicates that this effect is smaller for this system than for HOPO. This is mainly due to the fact that the ratio of the transition state height with respect to the frequency of the fundamental torsion is much larger (about 20) than for HOPO (about 6). Consequently, high lying correlation functions within the VCI calculations are less affected and thus the introduced approximation of two independent calculations appears to be justified.

### 4.2 *cis*‐Thioacrolein

The discussion of the results for *cis*‐thioacrolein mirrors to some extent that of *trans*‐thioacrolein. For example, the fundamentals of the CH‐stretchings $\nu_{2}$ and $\nu_{3}$ show extremely low leading VCI coefficients, that is, 0.370 and 0.334 (ae‐VCI), respectively. With that, the assignment of the states is somewhat arbitrary. For example, according to Table 3, there appears to be an inconsistency between the fc‐VCI and ae‐VCI transitions frequencies for $\nu_{3}$. However, an analysis of the ae‐VCI calculation showed that besides the assigned transition at 3031.4 ${\text{cm}}^{-1}$, a resonating state at 2975.6 ${\text{cm}}^{-1}$ with a leading VCI coefficient of 0.330 can be found. Likewise, a resonating state at 3031.1 can be seen within the fc‐VCI calculation. With that, it is essentially impossible and meaningless to assign a unique quantum number to these transitions. The values in our tables always refer to the transitions with the largest VCI coefficient, but this, of course, is very sensitive to any parameters of the calculation. Due to these strong correlation effects, the VPT2 results differ more strongly from the VCI results for the CH‐stretching modes than for all others.

The agreement of the calculations with the experimental values of Korolev and Baskir [2] for the fundamentals in the fingerprint region is very good. Most remarkably, all anharmonic calculations show consistently a positive anharmonicity for mode $\nu_{18}$, which indicates strong quartic contributions in the potential function. This is revealed by the large quartic force constant $f_{1111}$ within the quartic force field being employed in the fc‐VPT2 calculations, which has a value of 4805 ${\text{cm}}^{-1}$. This is in contrast to the *trans*‐conformer, for which this constant has a value of merely 253 ${\text{cm}}^{-1}$.

## 5 Rotational Spectra and Spectroscopic Constants

Besides studying the vibrational spectrum of thioacrolein, RVCI calculations for the simulation of the rotational spectrum of *trans*‐thioacrolein have also been performed. The knowledge of the vibrational spectrum readily reveals that hot bands will play a major role, which renders the calculations not only expensive, but also very sensitive. This sensitivity originates mainly from strong couplings caused by near degeneracies. For example, the overtone of the lowest torsional mode, that is, $2\nu_{18}$ at 280.1 ${\text{cm}}^{-1}$, is extremely close to the $\nu_{13}$ transition at 280.7 ${\text{cm}}^{-1}$, which are coupled by a type‐1 Fermi resonance. Likewise, the $3\nu_{18}$ overtone at 420.8 ${\text{cm}}^{-1}$ couples with the $\nu_{13}+\nu_{18}$ combination band at 423.7 ${\text{cm}}^{-1}$. The lowest eight vibrational states were found to show sizable hot band effects. Georgiou and Kroto [3] tabulated a reduced line list of rotational transitions for *trans*‐thioacrolein, and Cabezas et al. [4] provided a line list with 638 entries in the Supporting Information of their paper. In a first step, a comparison with these lists of experimental microwave data is provided, and the results are summarized in Table 4.

**TABLE 4:** Computed (ae) and refined rotational transitions (in MHz) of *trans*‐thioacrolein in comparison to experimentally determined data.

Transition	$\nu_{i}$ (ae)	Δ ^a^	$\nu_{i}^{\prime }$ (ae)	Δ ^a^	$\nu_{i}$ (exp)^b^	$\nu_{i}$ (exp)^c^
5 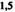 ‐4 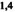	26754.459	2.436	26750.590	−1.433	26752.023	26733.804
5 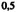 ‐4 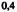	27150.781	3.998	27146.839	0.056	27146.783	27128.855
5 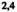 ‐4 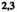	27159.175	4.066	27155.232	0.123	27155.109	27137.145
5 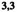 ‐4 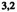	27162.173	4.359	27158.229	0.415	27157.814	27140.017
5 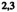 ‐4 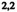	27168.169	4.168	27164.224	0.223	27164.001	27145.995
5 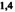 ‐4 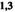	27561.493	5.789	27557.477	1.773	27555.704	27538.069
6 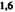 ‐5 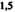	32103.912	2.794	32099.069	−2.049	32101.118	32079.242
6 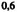 ‐5 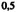	32575.781	4.713	32570.852	−0.216	32571.068	32549.543
6 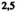 ‐5 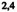	32590.171	5.021	32585.240	0.090	32585.150	32563.656
6 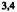 ‐5 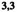	32595.267	5.414	32590.335	0.482	32589.853	32568.528
6 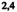 ‐5 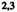	32605.760	4.892	32600.825	−0.043	32600.868	32579.319
6 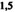 ‐5 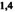	33072.233	6.746	33067.214	1.727	33065.487	33044.299
7 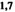 ‐6 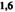	37452.765	3.265	37446.949	−2.551	37449.500	37423.973
7 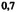 ‐6 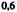	37998.083	5.444	37992.167	−0.472	37992.639	37967.530
7 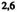 ‐6 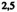	38020.567	5.861	38014.647	−0.059	38014.706	37989.634
7 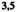 ‐6 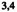	38028.661	6.307	38022.740	0.386	38022.354	37997.344
7 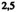 ‐6 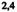	38045.749	5.850	38039.824	−0.075	38039.899	
7 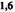 ‐6 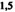	38582.373	7.829	38576.361	1.817	38574.544	38549.818

^a^ Deviation with respect to the data listed in column 6.

^b^ Taken from Ref. [3].

^c^ Taken from Ref. [4].

Note that all RVCI calculations make use of the PES obtained from ae electronic structure calculations and were restricted to the more important *trans*‐conformer. The agreement of the computed RVCI transitions (2nd column) with the experimental values of Cabezas et al. [4] (6th column) is good and considerably better than the experimental data of Georgiou and Kroto [3] (7th column). Maximum deviations of the RVCI results with respect to the more recent values of Cabezas et al. do not exceed 8 MHz. The corresponding RVCI line list with more than 4.5$\cdot 10^{6}$ entries is provided in the zenodo database, see the Data Availability statement. However, all transitions in this list were found to be slightly too high in energy, and thus, they have been improved even further by a simple linear least‐squares fit. A scaling factor of 0.999818 and a small offset of 1 MHz resulted in the values listed in column 4 of Table 4. The maximum deviation from the results of Georgiou and Kroto could thus be reduced to about 2.5 MHz and the MAD to 0.777 MHz.

**FIGURE 2:**
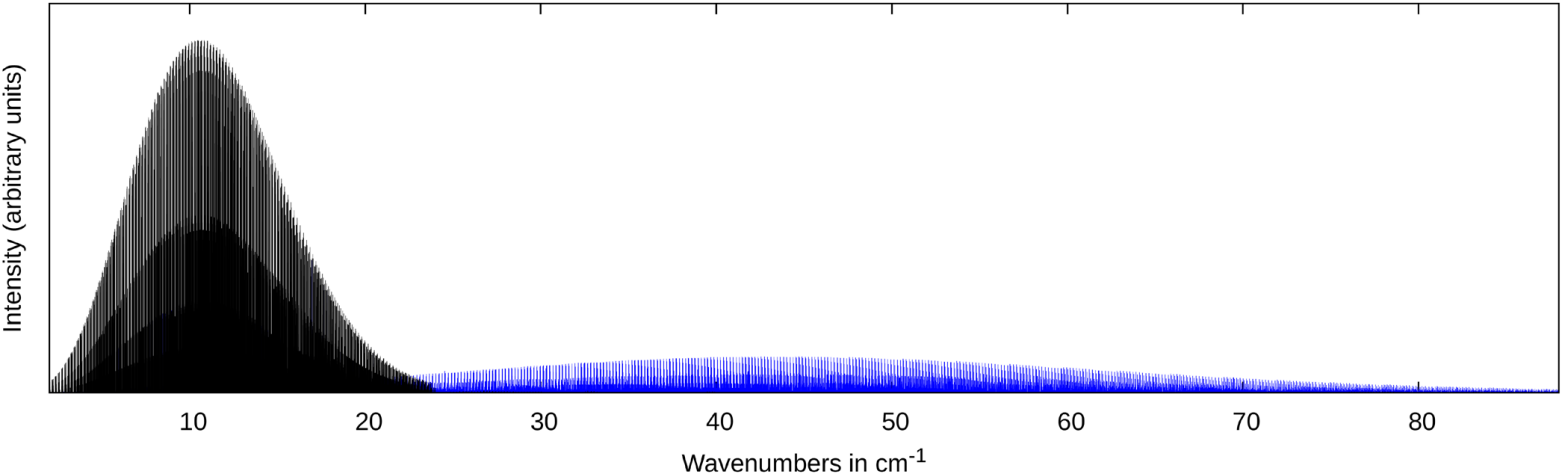
Simulated rotational stick spectrum of *trans*‐thioacrolein. Transition with $\Delta K_{a}=0$ are shown in black, those with $\Delta K_{a}=1$ in blue.

**FIGURE 3:**
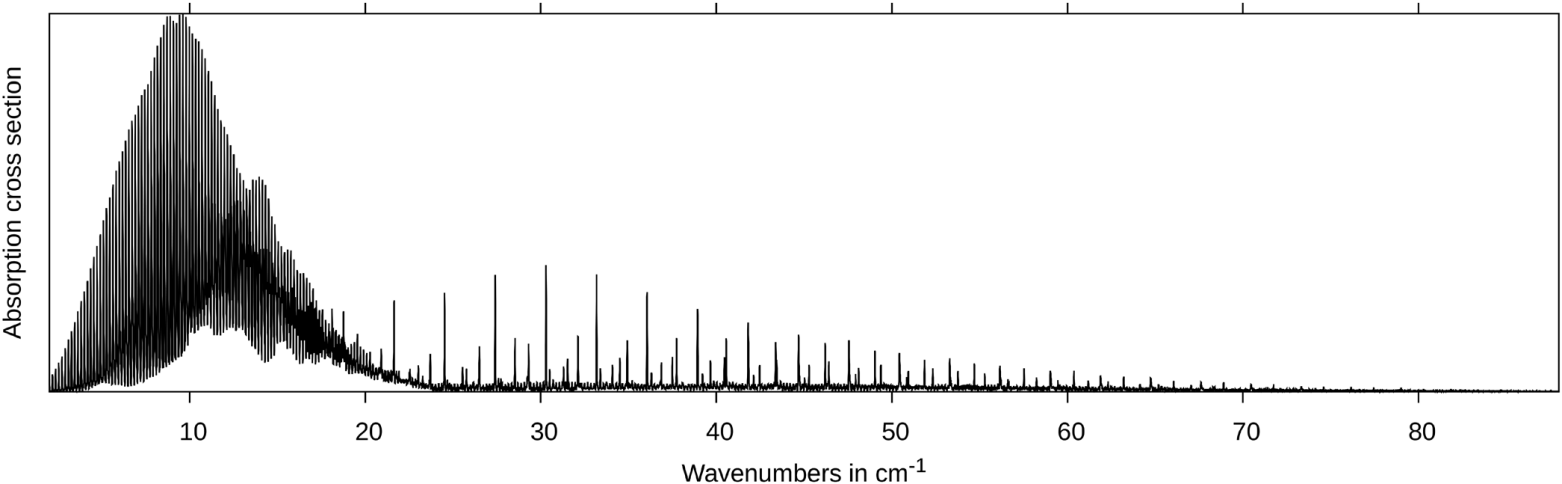
Rotational spectrum of *trans*‐thioacrolein including line broadening effects (FWHM = 0.01 ${\text{cm}}^{-1}$).

The vast amount of data in the line list is difficult to handle, and it is not trivial to extract physical insight from it. A visualization of the rotational spectrum is thus shown in Figure 2. Clearly, the spectrum is split into two regions, the first one ranging from 0‐23 ${\text{cm}}^{-1}$ and the 2nd from 23‐80 ${\text{cm}}^{-1}$. The first one, which is dominated by progressions of strong intensity, arises from transitions with $\Delta K_{a}=0$, while the 2nd range (marked in blue) refers to $\Delta K_{a}=1$ transitions. The same plot, including line broadening effects (Doppler broadening) by means of Gaussians with a FWHM of 0.01 ${\text{cm}}^{-1}$, is shown in Figure 3.

**FIGURE 4:**
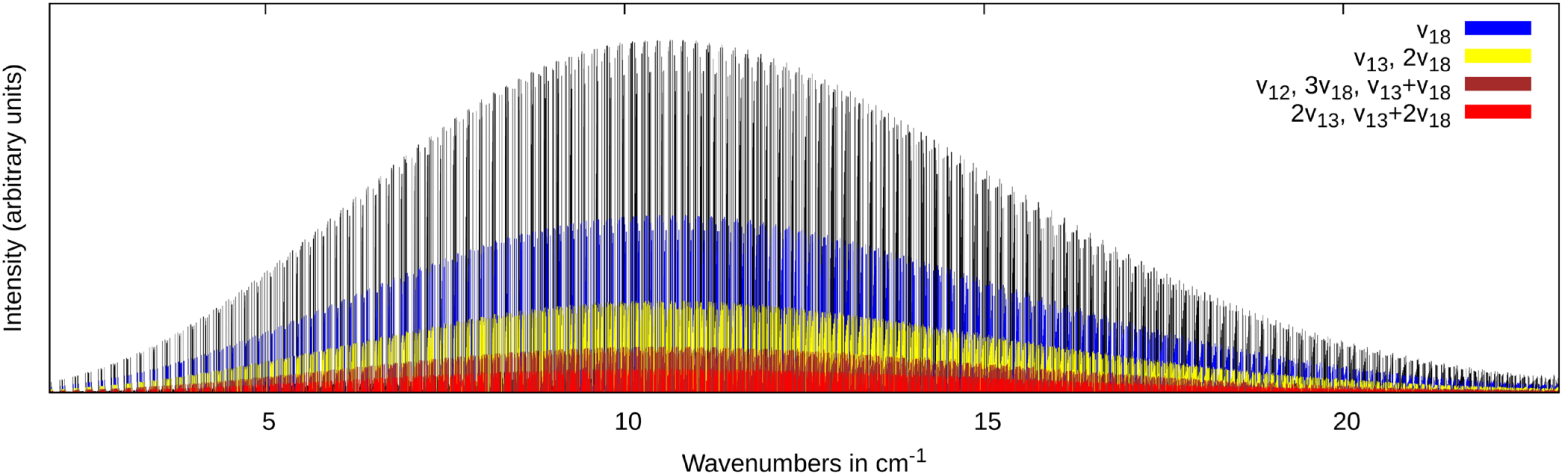
Fraction of the simulated rotational spectrum of *trans*‐thioacrolein. Hot band transitions are marked by color.

Of course, the FWHM value of 0.01 ${\text{cm}}^{-1}$ is somewhat arbitrary, and the spectrum will change upon variation of it, but the dominant features remain. While the spectrum beyond 20 ${\text{cm}}^{-1}$, including line broadening effects, is dominated by a progression of peaks, these are hidden behind some more intense rovibrational transitions in the stick spectrum. This progression arises from the Q‐branches in dependence on the K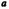 quantum number. It is the high density of transitions within the individual Q‐branches, which, in the case of line broadening, sum up to considerable absorption cross sections, which outperform the strong intensity of individual, but more isolated transitions, which shape the stick spectrum. This behavior is very similar to that observed for thiopropynal in a recent study [33]. Figure 4 shows the contributions of hot bands for the $\Delta K_{a}=0$ transitions in the most intense region of the spectrum.

Although eight vibrational states contribute to the hot band transitions, only four groups of them can be distinguished in Figure 4. This is due to the near degeneracies outlined above, which result in very similar populations of these states.

The visualization of the spectrum allows for an understanding of the individual progressions and couplings, but the agreed standard presentation of these data is in terms of spectroscopic constants, in which all this information is condensed to just a few parameters. Rotational constants, quartic and sextic centrifugal distortion constants as arising in Watson's S and A‐reduced Hamiltonians [34, 35, 36, 37] are summarized in Tables 5 and 6 for *trans* and *cis*‐thioacrolein, respectively.

**TABLE 5:** Spectroscopic constants^a^ of *trans*‐thioacrolein obtained for Watson's S and A‐reduced Hamiltonians in the $I^{r}$ axis representation.

S‐Reduction				A‐Reduction				
Par.	Unit	VPT (fc)	VPT (ae)	Par.	Unit	VPT (fc)	VPT (ae)	Exp.
$A_{0}$	MHz	45601.49	45784.08	$A_{0}$	MHz	45601.49	45784.08	45777.04
$B_{0}$	MHz	2783.71	2795.52	$B_{0}$	MHz	2783.71	2795.52	2795.99
$C_{0}$	MHz	2623.60	2634.69	$C_{0}$	MHz	2623.60	2634.69	2635.20
$D_{J}$	Hz	359.323	361.466	$\Delta_{J}$	Hz	361.611	363.769	370.108
$D_{JK}$	kHz	−6.564	−6.582	$\Delta_{JK}$	kHz	−6.578	−6.595	−7.072
$D_{K}$	kHz	374.478	376.472	$\Delta_{K}$	kHz	374.490	376.484	400.787
$d_{1}$	Hz	−28.999	−29.165	$\delta_{J}$	Hz	28.999	29.165	28.946
$d_{2}$	Hz	−1.144	−1.151	$\delta_{K}$	kHz	2.479	2.495	2.646
$H_{J}$	mHz	0.067	0.067	$\Phi_{J}$	mHz	0.067	0.070	0.070
$H_{JK}$	Hz	−0.002	−0.003	$\Phi_{JK}$	mHz	−0.762	−0.814	−3.711
$H_{KJ}$	Hz	−0.324	−0.324	$\Phi_{KJ}$	Hz	−0.330	−0.330	−0.120
$H_{K}$	Hz	6.202	6.233	$\Phi_{K}$	Hz	6.206	6.237	6.388
$h_{1}$	mHz	0.016	0.016	$\phi_{J}$	mHz	0.016	0.016	0.015
$h_{2}$	mHz	0.001	0.001	$\phi_{JK}$	mHz	0.970	0.982	2.48
$h_{3}$	mHz	0.201	0.202	$\phi_{K}$	Hz	0.596	0.600	0.579

^a^ ae results for *trans*‐C
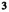
H
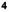
S: $A_{e}$ = 46276.66 MHz; $B_{e}$ = 2809.54 MHz; $C_{e}$ = 2648.73 MHz.

**TABLE 6:** Spectroscopic constants^a^ of *cis*‐thioacrolein obtained for Watson's S and A‐reduced Hamiltonians in the $I^{r}$ axis representation.

S‐Reduction				A‐Reduction			
Par.	Unit	VPT (fc)	VPT (ae)	Par.	Unit	VPT (fc)	VPT (ae)
$A_{0}$	MHz	17722.70	17774.76	$A_{0}$	MHz	17722.70	17774.76
$B_{0}$	MHz	3793.68	3812.42	$B_{0}$	MHz	3793.68	3812.42
$C_{0}$	MHz	3127.94	3139.50	$C_{0}$	MHz	3127.94	3139.50
$D_{J}$	kHz	1.652	1.649	$\Delta_{J}$	Hz	1.713	1.710
$D_{JK}$	kHz	−10.753	−10.687	$\Delta_{JK}$	kHz	−11.114	−11.048
$D_{K}$	kHz	63.661	63.558	$\Delta_{K}$	kHz	63.962	63.859
$d_{1}$	Hz	−422.617	−421.728	$\delta_{J}$	Hz	422.617	421.728
$d_{2}$	Hz	−30.110	−30.087	$\delta_{K}$	kHz	5.095	5.087
$H_{J}$	mHz	0.100	−0.115	$\Phi_{J}$	mHz	0.286	0.055
$H_{JK}$	Hz	0.017	0.020	$\Phi_{JK}$	Hz	0.030	0.032
$H_{KJ}$	Hz	−0.319	−0.331	$\Phi_{KJ}$	Hz	−0.363	−0.374
$H_{K}$	Hz	1.240	1.261	$\Phi_{K}$	Hz	1.272	1.292
$h_{1}$	mHz	0.199	0.118	$\phi_{J}$	mHz	0.219	0.137
$h_{2}$	mHz	0.093	0.085	$\phi_{JK}$	Hz	−0.001	−0.002
$h_{3}$	mHz	0.020	0.020	$\phi_{K}$	Hz	0.356	0.344

^a^ ae results for *cis*‐C
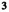
H
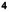
S: $A_{e}$ = 17847.96 MHz; $B_{e}$ = 3837.31 MHz; $C_{e}$ = 3158.28 MHz.

The agreement of the ae results with the experimental data of Cabezas et al. [4] for the rotational constants is excellent. The deviation for all three constants is less than 0.02%, while it is significantly larger for the frozen core results (0.44%). This is a well‐known effect and underlines the importance of the correction terms applied in the electronic structure calculations.

## 6 Summary and Conclusions

The vibrational and rotational spectra of thioacrolein have been studied by means of high‐level ab initio calculations. The basis for all these calculations was given by a multidimensional PES including up to 4‐mode coupling terms based on different levels of electronic structure theory ranging from explicitly correlated coupled‐cluster theory down to standard MP2 theory. Core‐correlation effects in combination with an estimate for high‐order excitations in the coupled‐cluster calculations were found to be important, in particular for the spectroscopic parameters and thus the rotational spectrum. Comparisons with experimental data have been provided whenever feasible, and very good agreement with these was found in most cases. Large deviations with respect to experimental results were observed for vibrational transitions in the CH‐stretching region, which arise from a (partial) loss of the state identity of these states in the VCI calculations and uncertainties in the experimental results. A number of reassignments have been provided for *trans*‐thioacrolein, while many predictions are provided for *cis*‐thioacrolein. Moreover, accurate estimates for the spectroscopic constants of *cis*‐thioacrolein are provided for the first time.

## Funding

This work was supported by Deutsche Forschungsgemeinschaft (Ra 656/29‐1, INST 40/575‐1 FUGG (JUSTUS2 cluster)).

## Conflicts of Interest

The author declares no conflicts of interest.

## Data Availability

The data that support the findings of this study, that is, potential energy surfaces and line list, are available online on the Zenodo database (https://www.doi.org/10.5281/zenodo.18376879).

## Endnotes

^1^
Accessed Jan. 23rd 2026; obtained from Clarivate (Web of Science). ©Clarivate 2026. All rights reserved.
